# Genotoxic evaluation of an industrial effluent from an oil refinery using plant and animal bioassays

**DOI:** 10.1590/S1415-47572010005000006

**Published:** 2010-03-01

**Authors:** Fernando Postalli Rodrigues, José Pedro Friedmann Angeli, Mário Sérgio Mantovani, Carmen Luisa Barbosa Guedes, Berenice Quinzani Jordão

**Affiliations:** 1 Departamento de Biologia Geral, Centro de Ciências Biológicas, Universidade Estadual de Londrina, Londrina, PRBrazil; 2 Laboratório de Fluorescência e Ressonância Paramagnética Eletrônica, Departamento de Química, Centro de Ciências Exatas, Universidade Estadual de Londrina, Londrina, PRBrazil

**Keywords:** *Allium cepa*, HTC cells, micronucleus, comet assay, petroleum waste disposal

## Abstract

Polycyclic aromatic hydrocarbons (PAHs) are genotoxic chemicals commonly found in effluents from oil refineries. Bioassays using plants and cells cultures can be employed for assessing environmental safety and potential genotoxicity. In this study, the genotoxic potential of an oil refinery effluent was analyzed by means of micronucleus (MN) testing of *Alium cepa*, which revealed no effect after 24 h of treatment. On the other hand, primary lesions in the DNA of rat (*Rattus norvegicus*) hepatoma cells (HTC) were observed through comet assaying after only 2 h of exposure. On considering the capacity to detect DNA damage of a different nature and of these cells to metabolize xenobiotics, we suggest the association of the two bioassays with these cell types, plant (*Allium cepa*) and mammal (HTC) cells, for more accurately assessing genotoxicity in environmental samples.

## Introduction

Genetic toxicology is a multidisciplinar field of research involved in detecting compounds capable of causing DNA damage and/or protecting DNA, with the aim of understanding potential biological consequences and molecular mechanisms of genetic material (Uhl *et al.*, 2003).

DNA noxious compounds are produced on a large scale by the secondary sector of world economy, this including the petrochemical industry through oil refining and related processes. This generally comprises heavy metals and organic compounds, as for example, mono- and polyaromatic hydrocarbons, and chlorinated and phosphorylated besides other contaminants, such as sulfuric acid (Houk, 1992; Ohe *et al.*, 2004). According to the Toxic Release Inventory (EPA) report of 2005, in the United States, the oil refining industry is one of 10 major sources emitting toxic chemicals into the environment.

Short-term biological assays have been widely used for evaluating environmental samples (Mitchelmore and Chipman, 1998; Avishai *et al.*, 2002; Klobucar *et al.*, 2003). By means of cytogenetic studies in higher plants, it is now possible to define chromosomal alterations and changes in *in vivo* cell proliferation, an example being the micronucleus test with *Allium cepa* developed by Evans *et al.* (1959). Thus, the clastogenic effect of polluted waters can be defined with considerable reliability (Ma *et al.*, 1995; Chandra *et al.*, 2005; Majer *et al.*, 2005; Srivastava *et al.*, 2005).

On the other hand, *in vitro* systems have proved to be more susceptible to the standardization of experimental conditions, availability and facile reproducibility of results (Rabello-Gay *et al.*, 1991). Its application provides for effective environmental sample analysis, such as the cytotoxicity assay employing fish primary epithelial cell cultures for defining the aquatic pollutants copper and prochloraz (Dowling and Mothersill, 2001), and the comet assay as applied to the RTH-149 fish hepatoma cell line (Avishai *et al.*, 2002). The comet assay itself appears to be an ideal tool in toxicological studies (Kuroda *et al.*, 1992; Avishai *et al.*, 2002), through being a rapid, simple, sensitive and relatively inexpensive method for detecting and analyzing DNA breaks in individual cells (Singh *et al.*, 1988; Speit and Hartmann, 1999; Brendler-Schwaad *et al.*, 2005).

There are few studies in which environmental samples have been tested by way of various assays simultaneously, thereby affording the means for comparing the sensitivity of different test-systems (Uhl *et al.*, 2003). Here, the genotoxicity of an effluent from an oil refinery was determined *in vivo* by the MN test in *Allium cepa*, and *in vitro* by the comet assay in HTC metabolizing cells. The results thus obtained were mutually compared.

## Materials and Methods

###  Cell line

HTC rat hepatoma cells were acquired from the Rio de Janeiro Cell Bank. Cells were grown in a DMEM/F-12 medium (Gibco), supplemented with 10% fetal bovine serum (FBS, Gibco) as monolayer in 25 cm^2^ flasks, in a BOD type incubator at 37 °C. Under these conditions, the doubling time was approximately 24 h for HTC.

###  Characterization of the effluent

The effluent from an oil refinery was used for testing. Both the removal of solids and colloids and the subsequent biological treatment were undertaken on the spot. Initial physic-chemical properties were: pH 6.25; dissolved oxygen = 6.20 mg O_2_/L; conductivity = 1242 μS/cm; temperature = 23 °C; total organic carbon = 20.07 ppm; and biochemical oxygen demand = 81.24 ppm. A complementary test was carried out to determine total phenols in the effluent by means of 4-aminoantipyrine in an alkaline medium and measuring absorbance in a FENTO model 482 spectrophotometer at 460 nm (AWWA, 1999).

Parameter temperatures, dissolved oxygen and water conductivity were continuously monitored throughout the experiments.

###  Micronucleus (MN) testing and determination of cell proliferation in *Allium cepa*

The method applied to MN testing in *Allium cepa* (common onion), with minor modifications, has already been described by Ma (1995). Prior to treatments, onion bulbs were cleaned to remove dried outer layers, washed in running water, and then partially submersed. Primordial root tissue was then allowed to grow for 48 h in a BOD type incubator at 25 ± 0.5 °C. Those displaying root growth of less than 2 cm were discarded. Sequentially, 3 bulbs were exposed for 24 h to each of the following effluent concentrations: 25, 50 and 100%. Well water without chlorine used to dilute the effluent was the negative control, whereas methylmethane sulfonate (MMS) dissolved in well water at 10 mg/mL, was the positive. During the entire assay, the bulbs were kept in the dark at a constant temperature of 25 °C. In each treatment, 5 randomly chosen roots from each bulb, and stained with aceto-chloride orcein according to the Tjio and Levan (1950) method, were used to prepare 5 slides. Subsequently, 5000 cells obtained from that portion of the root tip adjacent to the meristem (F1 generation of cells) were analyzed (1000 cells/slide). MN frequency was obtained by dividing the number of cells with MN by the number of cells analyzed in each treatment, and multiplying by 100. Furthermore, the mitotic index (MI) was defined by examining 5000 meristematic cells per treatment (1000/root), and was expressed as a percentage of cells in mitosis in all the evaluated meristematic cells.

###  Comet assay in hepatoma cells (HTC)

The comet assay was applied using *Rattus norvegicus* hepatoma cells (HTC), according to the protocol described by Uhl *et al.* (1999, 2000), and following the premise proposed by Tice *et al.* (2000). HTC cells were grown in a DMEM F12 medium for 24 h prior to treatment. Subsequently, the culture medium was discarded and the cells were washed with PBS (pH 7.4). All the treatments were applied directly to cells for 2 h, with three repetitions each. The experimental protocols were: a) treatments with a culture medium prepared with 25, 50 and 100% of effluent diluted with water, where the concentration of the medium (0.468 g DMEM-F12 per mL) was the same for all; b) positive control with benzo[a]pyrene added at 10 μg/mL in the culture medium; and c) negative control with the culture medium.

Finally, cells were harvested by trypsinization using 0.5 mL of 0.025% trypsin and 0.5 mL PBS (pH 7.4), whereupon a small sample was employed for testing cytotoxicity by the trypan blue exclusion method. A 20 μL aliquot of the cell suspension was embedded in low melting-point agarose (120 μL), and then spread onto a slide previously coated with normal melting-point agarose. Next, the cells were submersed in a lysis solution (1 mL of Triton X-100, 10 mL of DMSO and 89 mL of lysis stock solution, pH 10.0 - lysis stock solution: 146.1 g of NaCl (2.5 M), 37.2 g of EDTA (100 mM), 1.2 g of Tris (10 mM), ~8.0 g of solid NaOH, 890 mL of distilled H_2_O and 10 g of sodium lauryl sarcosinate), where they were left overnight. Electrophoresis was then carried out at pH > 13.0 (30 mL of 10 N NaOH, 5 mL of 200 mM EDTA - pH 10.0 and double-distilled H_2_O added to complete 1000 mL); gels were run for 20 min at 300 mA and ~0.8 V/cm, preceded by 20 min submersion in buffer to denature DNA. After electrophoresis, the cells were neutralized (48.5 g of Tris in distilled H_2_O to a final volume of 1000 mL and a final concentration of 0.4 M, pH 7.5), and then fixed in absolute ethanol (10 min). The slides were stained with ethidium bromide (0.02 mg/mL) at the moment of analysis, using a NIKON fluorescence microscope with a B-3A filter (excitation of λ = 520 nm) and 40 X objective.

Each treatment was independently repeated three-fold. In each case, 100 cells were examined microscopically (Kobayashi *et al.* 1995), and classified according to the following criteria: *class 0* - cells with undetectable damage, in which no tail was visible; *class 1* - cells with a tail shorter than the diameter of the nucleus; *class 2* - cells with a tail longer than and up to twice the diameter of the nucleus; and *class 3* - cells with a tail longer than twice the diameter of the nucleus. Apoptotic cells, wherein the nucleus was totally fragmented, were not taken into consideration in the analysis (Speit and Hartmann, 2005). The comet assay was carried out only in treatments in which cellular viability was greater than 80%, as determined by the trypan blue exclusion assay.

###  Statistical analysis of the data

The results obtained by comet assaying of HTC cells were evaluated by the Jandel Scientific Sigma Stat 2.0 program through analyzing the variance of means (ANOVA). The Kruskall-Wallis test (ANOVA), followed by the Tukey test for multiple comparison of means (α = 0.05 and α = 0.001), were used in the case of MN and MI data in *Allium cepa*.

## Results

###  Characteristics of the effluent - Determination of chemical and total phenolic constituents and physico-chemical parameters

The physico-chemical parameters of the effluents after periods of treatment and negative control were monitored throughout the experiment. Values (means ± SD) remained stable during experiments for conductivity, with the following results for control: temperature = 22.75 ± 0.5 °C; pH = 8.23 ± 0.04; dissolved oxygen = 7.94 ± 0.1 mg O_2_/L; and conductivity = 186.25 ± 4.65 μS/cm. The results with effluents were: temperature = 22.75 ± 0.5 °C; pH = 7.83 ± 0.14; dissolved oxygen = 7.71 ± 0.24 mg O_2_/L; and conductivity = 1957.33 ± 0.58 μS/cm. The concentration of total phenols in the effluent was 24 μg/L. Other organic compounds detected are shown in Table 1.

###  MN test in *Allium cepa*

The values obtained for the parameters analyzed in the root tip cells of *Allium cepa* are shown in Table 2. Effluent concentrations of 25, 50 and 100% did not induce the formation of MNs in F1 generation root cells at a frequency that differed significantly from the negative control. In relation to MMS results (positive control), the frequency of MNs in cells exposed to the effluent were significantly lower. Similarly, it was observed that there were no significant differences in mitotic index (MI) means values for any of the effluent concentrations tested versus negative control (p > 0.05).

###  Comet assay in HTC cells

**Figure 1:**
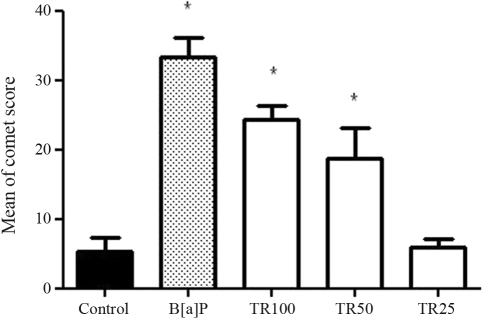
Mean scores of DNA damage in HTC cells. The values were obtained by summing up the number of cells identified in each class of damage and multiplying by the value of the particular class (from 1 to 3). The averages were calculated from reckoning 100 cells per treatment. TR100 = treatment with 100% of effluent; TR50 = treatment with 50% of effluent; and TR25 = treatment with 25% of effluent. B[a]p = Benzo[a]pyrene; control = water. * = significantly different in relation to control (p > 0.05).

The results obtained with this bioassay are presented in Figure 1. Higher concentrations (50 and 100%) caused an increase in DNA damage, whereas at 25%, there was no alteration in DNA migration patterns. Cell viability remaining higher than 90% in all treatments indicated non-cytotoxity in tested concentrations.

## Discussion

An oil refinery effluent was tested for its mutagenic and genotoxic potential through micronucleus (MN) testing in *Allium cepa* and comet-assaying in a rat hepatoma cell line (HTC)*.* As *in vitro* assays are proposed for the detection of environmental mutagenic agents (Kuroda *et al.* 1992), so bioassays with plants are also recommended for investigating compounds present in environmental samples and which cause genetic instability in DNA (Ma *et al.*, 1983; Plewa and Wagner, 1993; Rodrigues *et al.*, 1997). *Allium cepa* has proved to be an effective *in vivo* experimental model for determining both the toxic and genotoxic effects of substances and complex mixtures. On the other hand, HTC cells, due to their origin-tissue, are proficient in the metabolism of xenobiotics.

According to EPA (2005), in the United States, industries involved in refining petroleum products are among the most profuse emitters of toxic chemicals into the environment. Waste disposal therefrom has been shown to be highly toxic, often due to the soluble part, which contains inorganic (*e.g.*, salts and heavy metals) and organic (*e.g.*, oils, fats, PAHs and BTEX) compounds (Claxton *et al.*, 1998; Almeida-Val *et al.*, 2002). Chemical analysis of the effluent under study demonstrated the presence of various organic, including aromatic, substances, viz., toluene, ethylbenzene, xylene, pyrene, benzo[a]anthracene, benzo[a]pyrene and phenolic compounds. Although these were detected at lower concentrations than the limit allowed for the discharge of industrial effluents into water bodies (CONAMA - National Council of Environment [2005]. - Definition on water classification - CONAMA, 357. Brazil.), there are always risks associated with exposure at low concentrations (Ostergaard *et al.*, 2007).

The history of aromatic chemicals, as for example polyaromatic hydrocarbons (PAHs), demonstrates a relationship between the appearance of tumors in fishes and their presence in the water and sediment (Stein *et al.*, 1990; Myers *et al.*, 1991), thereby evinving their carcinogenic properties (Stalker *et al.*, 1991; Pacheco and Santos, 2001; White, 2002; Cebulska-Wasilewska *et al.*, 2005). Among the PAHs, nitropyrene and benzo[a]pyrene (B[a]p) are capable of forming DNA adducts after metabolic activation, this occuring in vertebrates (Roy *et al.*, 1989), more commonly in fishes, through their possessing a cytochrome P-450 system which is very active in this process (Smolarek *et al.*, 1987; Varanasi *et al.*, 1989; Sikka *et al.* 1991; Mitchelmore and Chipman, 1998).

Although considered one of the most potent mutagens, the PAHs encountered in the effluent studied were incapable of inducing MN formation in *Allium cepa* F1 cells, as was also demonstrated in other studies with plants (Veleminsky and Gichner, 1988). On the other hand, comet assaying indicated detectable DNA damage in HTCs. As it is known that PAH metabolism in humans and other mammals is complex (Scicchitano, 2005), and that HTCs are proficient in metabolizing these xenobiotics, this appears to be a likely explanation of the positive results observed.

The most important aspect in the understanding of these results appears to be that of PAHs acting indirectly on DNA. In other words, they need to be metabolically activated to be able to cause damage, this requiring the production of electrophilic metabolites by the cytochrome P450 system of phase I metabolism in the detoxification process (Cohen and Rice, 2001; Lee and Steinert, 2003). Thus, one must consider that higher plants also possess a cytochrome P450 system involved in detoxification processes (Werc *et al.*, 1990; Sanderman, 1992; Guencheva and Henriques, 2003), which would make *Allium cepa* an appropriate test organism for the analysis of the biotransformation of these chemical compounds. However, Plewa and Wagner (1993) reported that direct exposure to indirect-acting compounds in plants has proved to be of little effect. Related studies have raised the question as to whether plants can be used for detecting compounds that cause DNA damage and cancer in humans (Plewa and Wagner, 1993). This extrapolation depends directly on whether plants have a metabolizing system similar to that found in animals. However, Higashi (1988) and Sanderman (1992) found that plant cells contain active enzymes of the cytochrome P450 system, but at much lower activity levels than in rodents and humans. Moreover, their specific substrates differ substantially from those in mammals. Thus, the absence of mutagenic effects in *Allium cepa*, as observed in the present study, could be associated with the lower activity of the biotransforming system of xenobiotics in these root cells, as well as with the low quantity of aromatic compounds in the effluent. Uhl *et al.* (2003) observed the relative insensitivity of bioassays with plants to environmental contaminants, including PAHs, when these are dispersed in the environment and occur at relatively low concentrations.

Phenols were also present in the effluent in question. According to Spencer *et al.* (2007), these significantly increase the number of micronuclei in rats. Mitteregger *et al.* (2007) demonstrated toxicity in *Allium cepa* after exposure to environmental samples of water and sediment containing phenolic compounds. In the present study, on the contrary, even with an increase in MN in *A. cepa*, no toxic effects were revealed in cell proliferation assessment in the form of a decrease in cell growth (MI). On the other hand, Huang *et al.* (2007), by using the comet assay, also reported a positive and significant correlation between DNA damage in the liver of frogs (*Bufo raddei*) and the concentration of oil and/or phenol present from nearby petroleum plants. The DNA damage found in HTC cells could also be attributed to phenols present in the effluent. Further consideration should also be given to the monoaromatic compounds, benzene, toluene and ethylbenzene, detected in the effluent from the refinery under study. These were held responsible for the mutagenic effects detected in some cases reviewed by Ohe *et al.* (2004), when applying the Ames test, as well as other *in vitro* assays with bacterial, fungal, plant and animal cells. Likewise, these agents may have contributed to the *in vitro* genotoxic damage observed in the present study.

It is also common to find genotoxic agents in the form of heavy metals, in effluents from oil refining. In this study, the effluent was not assayed for these. Nevertheless, further investigation showed that there was induced DNA damage detected *in vivo* through comet assaying, and which gradually and rapidly underwent repair in its absence (data not shown), thereby implying no cellular accumulation of heavy metals. This observation further reinforces the notion of the preponderant role of effluent aromatic compounds in the genotoxic damage detected in HTC cells.

Nevertheless, according to Houk (1992), environmental samples should always be primarily evaluated as being a mixture of chemical compounds which may or may not interact with each other synergistically, additively or antagonistically, thereby generating a noxious effect.

An analysis of the present results also led us to consider that the diverse cellular responses obtained with comet assaying in HTC cells, as compared to MN testing in *Allium cepa* could even be attributed to the different end points contemplated (Guterres *et al.*, 2005). Comet-assaying detects DNA strand-breaks and alkali-labile sites, among other primary lesions that could be repaired (Tice, 2000). On the other hand, micronucleus testing detects lesions that have not been repaired, thereby becoming permanent after one cell-cycle (Rabello-Gay *et al.*, 1991), or rather, clastogenic effects that result in chromosomal breakage and/or aneugenic effects, thus terminating in alterations in chromosomal anchorage to the mitotic spindle. In the present study, there was insufficient time for the possible repair of lesions caused by the treatments in HTC cells, since the cells were harvested immediately after the exposure period and submitted at once to the comet assay protocol. Therefore, it is likely that primary lesions formed would be detected. However, in *Allium cepa* MN assaying it is believed that if the test substance produced DNA adducts, these lesions did not lead to those chromosomal breaks that give rise to micronuclei.

The results reinforce the idea that the use of various experimental protocols with regard to the type of assay, cell exposure time and the type of cells employed, can lead to a better determination of the mechanism of toxicity of a mixture (Kuroda *et al.*, 1992), since diverse mechanisms and parameters are involved in different genotoxicity tests (Zhong *et al.*, (2001).

Thus, although it is difficult to tell which of the chemical components identified and quantified in the effluent studied caused the detected damage, the results do indicate that the main genotoxic agents in this industrial wastewater are xenobiotic-metabolizing dependent aromatic hydrocarbons, which exerted no detrimental effect on the plant cells employed. If DNA lesions did in fact occur in these cells, they did not occasion chromosomal alterations leading to the occurrence of micronuclei in the F1 cells.

## Figures and Tables

**Table 1:** Mono-aromatic and poly-aromatic compounds present in the effluent.

Hydrocarbon	ppb^1^
Mono-aromatic	
Toluene	75
Ethylbenzene	126
Xylene	85
Poly-aromatic	
Pyrene	9
Benzo(a)antracene	33
Benzo(a)pyrene	61

^1^ppb = parts per billion.

**Table 2:** Occurrence of micronuclei in F1 cells and mitotic indices in *Allium cepa*.

Exposure (24 h)		Mean MN (% ± SD)	Mean MI (% ± SD)
	100%	1.6 ± 1	18.44 ± 1.5
Effluent	50%	1.4 ± 0.5	16.74 ± 3.5
	25%	1.4 ± 1	16.48 ± 1.8

Water		1.2 ± 0.5	17.74 ± 1.9

MMS		4.0 ± 1*	15 ± 3.9

Mean values: % in 5000 cells/treatment; MN = micronucleus; MI = mitotic index; SD = standard deviation. * = significant difference in relation to all the other samples (p > 0.05).
